# Remote skin self‐examination training of melanoma survivors and their skin check partners: A randomized trial and comparison with in‐person training

**DOI:** 10.1002/cam4.3299

**Published:** 2020-08-06

**Authors:** June K. Robinson, Racheal Reavy, Kimberly A. Mallett, Rob Turrisi

**Affiliations:** ^1^ Department of Dermatology Northwestern University Feinberg School of Medicine Chicago IL USA; ^2^ Biobehavioral Health and Prevention Research Center The Pennsylvania State University University Park PA USA

**Keywords:** melanoma survivors, remote training, skin self‐examination

## Abstract

**Background:**

Compared with other cancers, melanoma has the longest delays measured as the median time to patient presentation for care from symptom onset. Time to presentation for care is a key determinant of outcomes, including disease stage, prognosis, and treatment.

**Methods:**

Melanoma survivors with localized disease and their skin check partners enrolled in two sequential randomized control trials of skin self‐examination (SSE) training. In Phase 1, the pair read a workbook in the office and had quarterly total body skin examinations with a study dermatologist. In Phase 2, materials were mailed to pairs, whose surveillance was with a community physician. SSE knowledge, performance (frequency and extent), and identification of concerning moles were compared between phases.

**Results:**

Among 341 patients, 197 received the workbook and the others were controls. Knowledge in performing SSE was higher for the workbook relative to controls in both phases. The SSE frequency ranged from 2.38 to 5.97 times in 9 months. Patients randomized to the workbook in both phases performed significantly more SSE than controls at 9 months (*P *< .05). In both phases, trained survivors performed significantly more SSEs on the scalp than controls at 9 and 18 months (*P *< .05). Phase 1 survivors performed significantly more SSEs on the abdomen, buttocks, and soles of the feet than controls, but this did not occur in Phase 2. Finally, in both phases, survivors trained with the workbook resulted in greater detection of suspicious lesions and melanomas.

**Conclusions:**

These findings justify the benefits of remote SSE training for patients as an adjunct to provider‐administered screening.

## Graphical abstract

The flexibility of remote training allowed greater participation than in‐person training with scheduled skin examinations with the doctor. Common patient follow‐up burdens such as difficulties scheduling appointments, time away from work and family, transportation constraints, and the cost of the physician visit were reduced by remote education. Both types of training resulted in greater detection of suspicious lesions and melanomas by trained melanoma survivors than by controls. Figure A shows SSE of the abdomen, B SSE of the scalp.

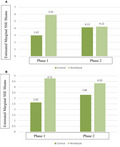

## 1 INTRODUCTION

In the United States (US), there will be an estimated 100,350 new cases of melanoma and 6,850 deaths in 2020.^1^ Melanoma increased by 270% between 1973 and 2002 in the US.^2^, ^3^ By 2030, the number of newly diagnosed cases is expected to more than double and the annual cost of treating newly diagnosed melanomas is estimated to triple from $457 million in 2011 to $1.6 billion in 2030.^2^, ^3^, ^4^ Time to presentation for care is a key determinant of the patient outcome,^5^ thus, early detection determines disease stage, prognosis, treatment, and cost to the payer. Compared with other cancers, melanoma has the longest delays measured as the median time from symptom onset to patient presentation.^6^ Until, melanoma early detection is effectively self‐managed by people at‐risk to develop melanoma, people will present for care with advanced melanoma and incur significant mortality, morbidity, and health care costs.

Since most melanomas are visible on the surface of the skin at a curable phase in their evolution, people can check their moles with the assistance of a skin check partner. Teaching at‐risk patients to check their skin has been hampered by lack of easily disseminated effective interventions; therefore, we developed a structured skin self‐examination (SSE) training intervention (Mole Score™). In the first phase of our research (Phase 1) with melanoma survivors (patients) and their skin check partners (partners), a randomized control trial of partner‐assisted SSE training was performed during an office visit and reinforced during quarterly total body skin examinations with a study dermatologist for two years. Our SSE training program has shown evidence that: (a) it can be implemented with fidelity in the clinical office in a variety of formats (structured face‐to‐face discussion, workbook, and tablet personal computer); (b) it results in both short‐ and long‐term maintenance of SSEs; and (c) it results in accurate evaluations of concerning lesions relative to dermatologists’ skin examinations.^7^, ^8^, ^9^, ^10^, ^11^ Although training patients and partners to perform SSE during clinic visits demonstrated their capacity to learn and perform them, the approach was not easily implemented or cost effective for wide scale dissemination; therefore, remote SSE training was needed. In the second phase (Phase 2), materials from Phase 1 were mailed to patients and their partners, who were randomized to control or remote training with mailed materials. Phase 2 patients received their surveillance from community physicians.

The unique opportunity to examine the effect of in‐person and remote training with the same materials was explored by comparing the two sequential research phases for differences in the following outcomes: SSE knowledge, performance (frequency and extent), and identification of concerning moles and melanomas. Although the two research phases were not conducted concurrently, exploring outcomes between participants randomized to control or workbook training in the two phases may provide guidance about the feasibility and effectiveness of remote patient SSE training.

## 2 MATERIALS AND METHODS

### 2.1 Patient selection

Patients with a history of melanoma in the past year were recruited using Enterprise Data Warehouse (EDW) of Northwestern University, which is a repository of patients willing to participate in research obtained by searching the electronic health records of Northwestern Medicine. Patients with melanoma and their partners were eligible if (a) both were 21 to 80 years old and had acceptable vision, b) patients had a diagnosis of stage 0 to IIB melanoma, (c) the pathology report confirmed the diagnosis, and (d) at least 6 weeks had elapsed since surgical treatment. Exclusion criteria were: (a) being overburdened with other comorbid diseases, (b) having a history of stage III or greater melanoma, or (c) being unable to commit to having skin examinations by the study dermatologist (JKR) every 4 months for 2 years (Phase 1) or to complete online surveys at 9 month intervals for 18 months (Phase 2). The Institutional Review Board of Northwestern University approved both phases of the research. Both melanoma survivors and their skin check partners provided written informed consent, and each received $20 to complete each survey in Phase 1 and $25 in Phase 2.^12^ Patients and partners completed separate surveys and were asked not to discuss their responses with each other.

### 2.2 Randomization

From June 2016 to April 2017, participants in Phase 1 were invited to enroll in Phase 2 and remained in their original group as controls or workbook intervention. Workbook participants from Phase 1, who enrolled from July 2011 to March 2013 and had ceased performing SSE at the end of Phase 1 participation (July 2103‐March 2015) were eligible to enroll in Phase 2. The random number sequence for newly enrolled Phase 2 subjects was generated as 2 controls to 1 workbook. In Phase 1, the recruiting personnel and participants were masked to the randomization until it was time to provide the intervention. At subsequent visits in Phase 1, a research assistant and the dermatologist were both masked as to which intervention the participants received. In Phase 2, all research personnel were masked to the randomization.

### 2.3 Intervention

In Phase 1, patients and partners read a 34‐page color workbook during an office visit, and received scorecards to record monthly scores of concerning moles for 24 months (a diary), a booklet of body diagrams to locate concerning moles, a lighted magnifying lens and a millimeter (mm) ruler.^13^ To assist the pair in identifying change in a mole, a scoring system was used to categorize the border, color, and diameter as 1 if normal, 2 if not sure, and 3 if abnormal. For example, the border was normal if it was smooth (score 1), if not sure (score 2), and abnormal or irregular if it contained jagged pointed projections extending from the mole into the surrounding skin (score 3). Similarly, the color was normal if 1 or 2 colors were uniformly distributed over the surface of the mole (score of 1), and abnormal if many colors (brown, black, blue, pink, white, and gray) with nonuniform color distribution (score 3). Last, the diameter was measured across the widest part of the mole and given a score of 1 if it measured 1 to 4 mm, score 2 for 5 mm, and score 3 for 6 mm or more.^14^, ^15^ To assist the pairs in making management decisions, they were told to monitor features scored as 2 (not sure) for change (evolution) at the next monthly SSE, and if change was noticed then pairs were told to notify the study dermatologist, who would evaluate the mole within 2 weeks. Change in the border or color of a concerning mole was usually identified after a median of 8‐9 months of study participation (standard deviation [SD] 2 months) of SSE by melanoma survivors and their skin check partners and the accuracy was confirmed by the dermatologist during total body skin examinations.^7^ Change in the diameter was often identified after a median of 12 months (SD 3 months) of study participation.

In Phase 2, online assessments self‐reported performing SSE in the preceding 9 months for 18 months. In Phase 2, all pairs randomized to receive the intervention were mailed the SSE workbook, booklet of body maps and scorecards, and kit consisting of a mm ruler and a lighted magnifying lens after the baseline survey was completed. Pairs confirmed receipt of materials by email. At the end of the Phase 2 study, pairs randomized to the control condition received the intervention materials by mail. In Phase 2, recommendations about seeking health care were based upon the cumulative score of three features of the mole (Border, Color, and Diameter) as follows: 3 = benign, stop checking the mole; 4‐7 = check the mole in one month; and 8‐9 = make an appointment with a health care provider (HCP) to have the mole checked in about 2‐3 weeks.

### 2.4 Follow‐up

In Phase 1, pairs had an in‐person visit every 4 months during which the self‐report survey was completed in the office, the pair reported if a visit with a physician for a concerning mole occurred since the last visit. The pair indicated if a skin biopsy was performed and gave authorization to obtain the results of the skin biopsy. If a biopsy was performed, the pair selected who found the lesion that was biopsied (eg, the patient, the partner, or the physician). A complete cutaneous skin examination was performed by the dermatologist. Biopsy specimens interpreted as melanoma were independently reviewed by two dermatopathologists during the 24 months of participation. In order to be entered into the database as a melanoma, the dermatopathologists needed to concur.

In Phase 2, pairs completed online baseline (prior to randomization), 9, and 18‐month surveys reporting their SSE practices in the preceding 9 months. The surveys used in both phases assessed SSE frequency and extent, partner assistance, anxiety associated with SSE, a history of seeking care for a concerning mole with a community physician, and who found the mole. If the biopsy was performed by a physician not affiliated with Northwestern Medicine, then, authorization to contact the physician for the pathology was obtained and the specimen was reviewed by a dermatopathologists at Northwestern Medicine. If the Northwestern Medicine dermatopathologists concurred with the diagnosis of melanoma, it was entered into the database. The Northwestern Medicine electronic health record (EHR) was searched for care of moles for all enrolled participants from baseline through 24 months. The EHR review extended for 6 months beyond the 18‐month self‐report survey to give participants the opportunity to have routine annual follow‐up with their HCPs. Thus, HCP’s could detect concerning moles that the participant may have failed to recognize.

### 2.5 Measures

The previous literature provided the measures.^7^, ^15^, ^16^ Patients and partners completed all surveys. Patient responses were used in this analysis because the past high concordance between patient and partner for SSE frequency and extent and knowledge was affirmed in 25 randomly selected responses from patients and partners at each survey.^11^ Responses to knowledge [16 items, eg, A smooth border is cause for concern [false]) were coded as 0 (incorrect) or 1 (correct)] and attitudes (7 items, eg, importance of SSE, I am at risk of developing a melanoma) used Likert scales with 1 (strongly disagree) to 5 (strongly agree). For analytic purposes, the 16 knowledge items and 7 attitudes items were averaged to create composite scores for each construct. Because these scales assessed SSE for the months prior to the survey at different phases of the study, for example, 8 and 16 months for Phase 1 and 9 and 18 months for phase 2, they are referred to as short‐ and long‐term follow‐ups, respectively.

Based upon the responses of Phase 1 participants, skin areas were determined to be easy to see alone, hard to see alone, and sexually sensitive.^16^ Among the locations with ease of self‐checking (face, front of neck, chest, arms, hands, abdomen, and front of thighs), the abdomen was the location selected by participants as the most representative of deliberate skin checks. Participants identified the need for partner assistance in checking areas not deemed sexually sensitive as the following areas: scalp, ears, back of neck, back and shoulders, back of thighs, and soles of feet. The scalp and soles of feet were the greatest burden to the partner to check. The sexually sensitive area, which both genders accepted the need to check because it may have had sun exposure, was the buttocks. For brevity, differences between locations requiring or not requiring partner assistance are reported for the abdomen, buttocks, scalp, and soles of feet only. A mean score of the number of SSEs over the preceding months and the locations checked in Phases 1 and 2 was created.

### 2.6 Statistical analysis

**FIGURE 1:**
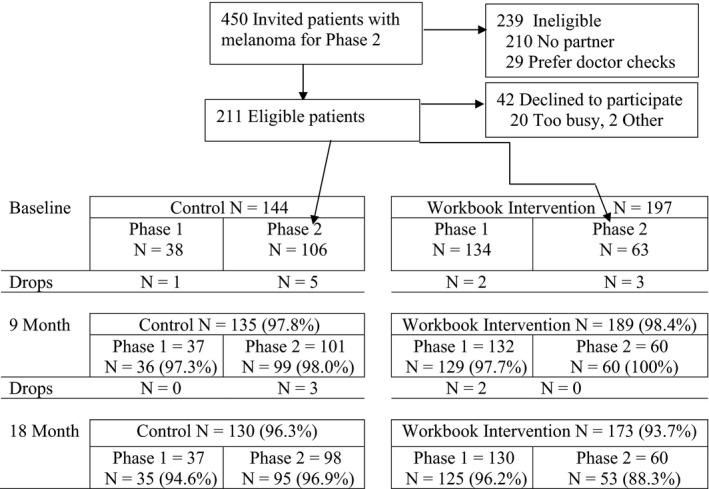
CONSORT Study Flow Diagram. CONSORT indicates Consolidated Standards of Reporting Trials

For total and subgroup arm sample sizes at each wave used in the analyses see CONSORT Study Flow Diagram (Figure 1).

*Power*. The sample size of 384 pairs was chosen based on an estimated 20% attrition over the duration of Phases 1 and 2 (we observed <10% average attrition for the four arms across the study). For comparison of Phase 1 and 2, it was determined that we would be able to detect effect sizes that correspond to small eta squares in the range of 2%.

*Analytic methods*. A series of 2 (Controls, Workbook) X 2 (Phase 1, Phase 2) ANOVAs were conducted on patients’ SSEs for abdomen, buttocks, scalp, and soles of feet. Second, for analysis of knowledge scores a 4 (Phase 1 Controls, Phase 1 Workbook, Phase 2 Controls, Phase 2 Workbook) X 3 (baseline, short, and long follow‐ups) mixed ANOVA was conducted. Tukey's tests were used for all post hoc follow‐up comparisons. All analyses were two‐tailed tests.

## 3 RESULTS

### 3.1 Description of the population

There were no significant differences in the demographic characteristics of sex, age, education, marital status, or income among the Phase 1 and 2 melanoma survivors randomized to control or workbook training (Table 1). There was a significant difference in the distance of the household from the medical center with Phase 1 participants living within 25 miles and Phase 2 living more than 25 miles (*P* = .02). Enrollment in Phase 1 was 57% (494 of 856)^11^ and in Phase 2 was 80% (169 of 211). Retention in the Phase 1 workbook training intervention was 59.7% (95 of 159) and in Phase 2 was 96.4% (190 of 197). (Figure 1.)

**TABLE 1:** Demographic characteristics of population Phase 1 (re‐enrolled in Phase 2) and Phase 2 (newly enrolled in Phase 2)

	Phase 1 Control (n = 38) (%)	Phase 1 Workbook (n = 134) (%)	Phase 2 Control (n = 106) (%)	Phase 2 Workbook (n = 63) (%)	Statistical Test
Sex					χ^2^(3) = 5.79, *P *> .05
Male	15 (39.4)	66 (49.3)	51 (48.1)	23 (36.5)	
Female	23 (60.5)	68 (50.7)	55 (51.8)	40 (63.5)	
Age (y)					ANOVA F(3) = 1.64, *P *> .05
18‐29	2 (5.3)	5 (3.7)	4 (3.8)	4 (6.3)	
30‐39	3 (7.9)	16 (11.9)	11 (10.3)	11 (17.4)	
40‐49	8 (21.0)	21 (15.7)	23 (21.7)	12 (19.0)	
50‐59	10 (26.3)	34 (25.4)	28 (26.4)	14 (22.2)	
60‐69	9 (23.7)	36 (26.9)	27 (25.4)	17 (26.9)	
70+	6 (15.7)	22 (16.4)	13 (12.2)	5 (4.7)	
Education					χ^2^(12) = 7.35, *P* > .05
Some High School or Less	0 (0.0)	2 (1.5)	1 (1.0)	0 (0.0)	
High School Graduate	2 (5.3)	4 (3.0)	3 (2.8)	1 (1.0)	
Some Post‐High School Education	4 (10.5)	16 (12.0)	10 (9.4)	9 (8.5)	
College Graduate	16 (42.1)	58 (43.6)	44 (41.5)	28 (26.4)	
Graduate Degree	16 (42.1)	53 (39.8)	48 (45.2)	25 (23.6)	
Marital Status					χ^2^(15) = 23.48, *P* > .05
Married	30 (78.9)	113 (84.3)	87 (82.0)	50 (79.4)	
Never Married	4 (10.5)	4 (3.0)	9 (8.5)	7 (6.6)	
Divorced	3 (7.9)	6 (4.5)	7 (6.6)	3 (2.8)	
Widow(er)	1 (2.6)	2 (1.5)	3 (2.8)	3 (2.8)	
Income					ANOVA F(3) = 0.74, *P* > .05
<$10 000	0 (0.0)	1 (0.8)	3 (2.9)	1 (1.0)	
$10 000‐$19 999	1 (2.6)	0 (0.0)	2 (1.9)	0 (0.0)	
$20 000‐$34 999	3 (7.9)	7 (5.3)	6 (5.7)	2 (1.9)	
$35 000‐$50 999	4 (10.5)	6 (4.5)	11 (10.3)	7 (6.6)	
$51 000‐$100 000	12 (31.5)	41 (31.1)	18 (16.9)	15 (14.4)	
$100 000+	18 (47.4)	77 (58.3)	66 (62.3)	38 (35.8)	
Domicile (miles)					χ^2^ (3) = 7.84 *P* = .02
<25	16 (42.1)	79 (58.9)	18 (16.9)	12 (11.3)	
26‐50	22 (57.9)	54 (40.3)	53 (50.0)	34 (32.0)	
>51	0	1 (0.7)	35 (33.0)	17 (16.0)	

### 3.2 SSE importance

There was no significant difference at baseline between Phases 1 and 2 for control or workbook intervention participants for the following variables assessed with Likert scale 1‐5: I am at risk of developing a new melanoma (range 4.39‐4.59), Importance of SSE (range 4.74‐ 4.78), It is very important for me to know the difference between a melanoma and other types of moles (range 4.78‐4.79), and I am very concerned about developing a new melanoma at some point in my life (range 4.21‐4.76). (One‐way ANOVA controlling for age for each variable *df* 2,338, p ranges from 0.249 to 0.718).

### 3.3 SSE knowledge

Analysis of the knowledge composite scores across the three waves for the different groups and phases resulted in a significant omnibus interaction (F[6, 628] = 2.85, *P* < .05). Follow‐up Tukey's HSD post hoc test analyses revealed several important trends (Table 2). First, no significant baseline differences were observed across all comparisons. Second, at 9 months significant differences in knowledge were observed between workbook and controls in Phase 1. Workbook recipients in Phase 2 did not have a statistically significance increase in knowledge.

**TABLE 2:** Comparison of knowledge for Phases 1 and 2

	Knowledge				
	Phase 1 Control Mean (SD)	Phase 1 Workbook Mean (SD)	Phase 2 Control Mean (SD)	Phase 2 Workbook Mean (SD)	Statistical test Mixed ANOVA
Baseline	0.73^a1^ (0.01)	0.75^a1^ (0.01)	0.75^a1^ (0.01)	0.75^a1^ (0.01)	F(6,628) = 2.85, *P *< .05 Tukey's CD = 0.03
9‐month follow‐up	0.76^a1^ (0.01)	0.80^b2^ (0.01)	0.76^a1^ (0.01)	0.78^a1^ (0.01)	
18‐month follow‐up	0.77^a1^ (0.01)	0.78^a1^ (0.01)	0.75^a1^ (0.01)	0.78^a1^ (0.01)	

Notes

Uncommon letter superscripts within an analysis reflect significant mean differences by Tukeys post hoc comparisons across the groups.

### 3.4 SSE frequency

**FIGURE 2:**
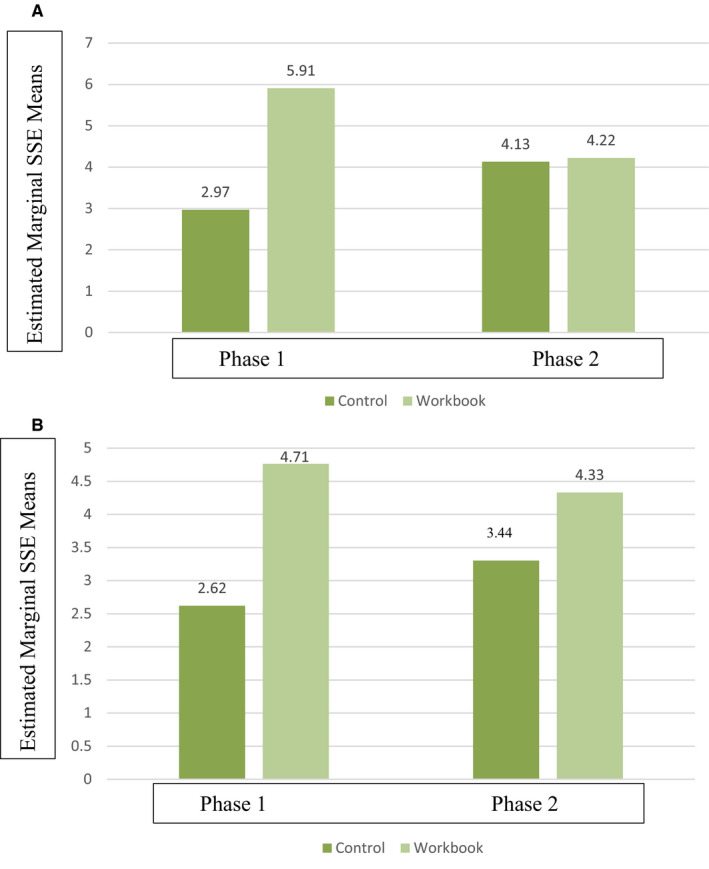
Skin self‐examination frequency of the A) abdomen and B) scalp in controls and melanoma survivors receiving the workbook in the prior 9 months

The frequency of SSE ranged from 2.38 times to 5.97 times in the preceding 9 months. None of the participants performed monthly SSE. Follow‐up comparisons showed the workbook group at 9 months checked significantly more SSE than controls for all locations in Phase 1 (*P* < .05) (Table 3). Checking the scalp was significantly different in both phases for workbook and controls at both 9 and 18 months [F(1,380) = 4.06, *P* = .045 for 9 months and F(1,332) = 3.84, *P* = .047]. (Figure 2B) Differences between workbook and controls on checking the abdominal location varied based on the phase of the study (F = 21.23, *P *< .01) (Figure 2A).

**TABLE 3:** Differences in skin self‐examination frequency by body part for Phase 1 and 2

	9‐month follow‐up Mean (SD)			18‐month follow‐up Mean (SD)		
	Control	Workbook		Control	Workbook	
Abdomen						
Phase 1	**2.97** ^a^ **(2.65)**	**5.91** ^b^ **(2.60)**	F(1,380) = 21.23, *P* < .001	**4.00** ^a^ **(3.10)**	**5.97** ^b^ **(2.83)**	F(1,332) = 7.45, *P* = .007
Phase 2	**4.13 (3.47)**	**4.22** ^c^ **(3.01)**	Tukey CD = 0.95	**3.66** ^a^ **(3.26)**	**3.77** ^c^ **(3.10)**	Tukey CD = 1.00
Buttocks						
Phase 1	**3.01** ^a^ **(3.01)**	**5.12** ^b^ **(2.87)**	F(1,380) = 5.11, *P* = .024 Tukey CD = 0.97	3.77 (3.16)	5.44 (3.02)	F(1,332) = 2.69, *P* > .05
Phase 2	**3.30** ^a^ **(3.09)**	**3.98** ^a^ **(2.94)**		3.22 (3.07)	3.77 (3.06)	
Scalp						
Phase 1	**2.62** ^a^ **(2.51)**	**4.71** ^b^ **(2.88)**	F(1,380) = 4.06, *P* = .045	**3.61 (2.85)**	**4.80 (3.00)**	F(1,332) = 3.84, *P* = .047
Phase 2	**3.44** ^a^ **(2.94)**	**4.33** ^b^ **(2.81)**	Tukey CD = 0.91	**3.63 (3.16)**	**4.86 (3.04)**	
Soles of Feet						
Phase 1	**2.38** ^a^ **(2.41)**	**4.52** ^b^ **(3.02)**	F(1,380) = 6.46, *P* = .011	3.47 (3.08)	4.80 (3.16)	F(1,332) = 1.43, *P* > .05
Phase 2	**3.28** ^c^ **(3.14)**	**3.87** ^c^ **(2.83)**	Tukey CD = 0.94	3.01 (2.96)	3.53 (2.90)	

Note

SD = standard deviation; bold values indicate a significant interaction present.

Uncommon superscripts within an analysis reflect significant mean differences by Tukeys post hoc comparisons.

### 3.5 SSE anxiety

There was no significant SSE induced anxiety among workbook training intervention participants in both phases over the time of active participation as assessed by responses to the following items: I feel in control of my health (F(1,151) = 1.34, *P *> .05), I experience upsetting memories of having a melanoma (F(1,151) = 0.07, *P *> .05), and I feel comfortable discussing my feelings with my skin check partner (F(1,151) = 1.42, *P *> .05). There was a marginal effect among intervention participants in Phases 1 and 2 of more positive feelings over time to: I feel I am doing something positive for my health (F(1,155) = 4.05, *P *=. 046).

### 3.6 Biopsies

In both phases, there was a significant difference between the workbook and control groups on whether the mole was biopsied with more moles biopsied in the workbook group χ^2^(1) = 8.58, *P* < .01. This indicated that melanoma survivors in the workbook group appropriately sought care for concerning moles.

### 3.7 Detection of melanoma

During Phase 2, 27 melanomas were detected among 341 subjects identified by EHR review for 24 months. Thus, 7.9% of participants developed a second melanoma. While the sample was small, there was a trend for participants receiving the workbook to detect melanomas at an earlier stage than controls in both phases (Table 4). When comparing the two phases for who detected the melanoma, there was not a significant difference for self‐detected at baseline, χ^2^(1) = 0.00, *P *> .05; 9 months, χ^2^(1) = 2.20, *P *> .05; or 18 months, χ^2^(1) = 2.01, *P *> .05. There was a significant difference for partners' detection at 9 months, χ^2^(1) = 4.82, *P* < .05 and 18 months χ^2^(1) = 4.05, *P* < .05 with Phase 2 workbook partners finding more melanomas than Phase 2 patients. Furthermore, the locations of the melanomas detected by partners were often in places that a person cannot easily see when alone, for example, scalp and back. In Phase 1, 6 of 21 (28.6%) partner detected melanoma were on the scalp, and in Phase 2, 5 of 13 (38.5%) melanomas were on the scalp. There was not a significant difference between the phases on “doctor” as the melanoma identifier at either baseline, χ^2^(1) = 0.04, *P *> .05; 9 months, χ^2^(1) = 0.02, *P *> .05; or 18 months χ^2^(1) = 0.03, *P *> .05.

**TABLE 4:** Melanoma Identification by Patient, Partner or Physician^a^

Identifying Person	Stage 0	Stage 1	Stage 2
Phase 1	(n = 28)	(n = 10)	(n = 0)
Workbook (n = 159)			
Patient	11	1	0
Partner	17	4	0
Physician	0	1	0
Control (n = 99)			
Patient	0	0	0
Partner	0	0	0
Physician	0	4	0
Phase 2	(n = 15)	(n = 10)	(n = 2)
Workbook (n = 194)			
Patient	5	1	0
Partner	10	3	0
Physician	0	0	0
Control (n = 151)			
Patient	0	1	0
Partner	0	0	0
Physician	0	5	2

^a^ Electronic health record review for 24 months.

## 4 DISCUSSION

While SSE performance was not quite equivalent between mailing the workbook to melanoma survivors with follow‐up care by the survivor's community physician and delivering the workbook in the office with periodic skin examinations by the study dermatologist, SSE performance and detection of melanomas were greater in both phases for trained melanoma survivors than for controls. Most pairs performed SSE every 3‐4 months. In the initial 9 months, there was a significant difference in knowledge between workbook and control participants in Phase 1 and a trend for increasing knowledge among workbook participants in Phase 2. In both phases, anxiety related to SSE training did not increase during the months of participation.

Partners, who received workbook training, checked hard to see locations, such as the scalp. Since patients with melanoma of the scalp died 1.84 times (HR, 1.84, 95% confidence interval 1.62‐2.10) more than any other location,^17^ partners’ engagement in checking the scalp may result in detecting thinner melanomas with improved survival. The incidence of a second melanoma in Phase 2 was 7.9%, which is like other US studies in which the percentage of melanoma patients developing a second primary melanoma was 7%‐12%.^18^, ^19^, ^20^, ^21^, ^22^, ^23^ (make this ref 18‐23) In contrast to breast, colorectal, or lung cancers, where provider‐administered screening is essential for early detection, people can identify concerning lesions on their own skin^24^; therefore, giving at‐risk people evidence‐based SSE training may improve early detection of melanoma.

Melanoma survivors more readily enrolled in the remote training of Phase 2 (80%) than in the in‐person training of Phase 1 which required periodic skin examinations (57%).^11^ Common patient follow‐up burdens such as difficulties scheduling appointments, time away from work and family, transportation constraints, and the cost of the physician visit were reduced by remote training. In an Australian study, one‐third of melanoma survivors with localized disease preferred fewer scheduled clinic visits in favor of relying on patient detection of melanoma with ready access to physicians if a concerning lesion is detected.^25^ Acceptance of SSE by melanoma survivors in this study may have been enabled by the assurance that if a concerning mole was detected a study physician would assess the mole within 2 weeks. While some melanoma survivors may prefer face‐to‐face physician visits, most Phase 2 participants learned SSE from reading and referring to the workbook and performed SSE with their skin check partner.

An important strength of this study was restricting enrollment in Phase 2 to those who had treatment for a melanoma within the last year (ie, an inception cohort). There are several limitations in our study. Recall bias may have caused overestimation of the frequency of SSE. Allowing Phase 1 workbook participants, who did not perform SSE after completing the two years of the study, to participate in Phase 2 may have contributed to the lack of improvement in knowledge in Phase 2.

In no instance, did the findings show poor SSE performance by individuals trained by the workbook. Thus, the findings of these two phases of research justify the benefit of structured SSE training for patients as an adjunct to provider‐administered screening. Personnel and material costs may prohibit physicians from mailing materials to patients. In the future, smartphone apps based on this workbook may instruct melanoma survivors how to perform SSE. Remotely enhancing SSE performance may require interactive apps that allow the patient to send images of concerning lesions to dermatologists or other HCPs and receive recommendations. At‐risk consumers appear to be ready to accept interactive apps.^26^ When the quality of images of concerning lesions taken by users supports diagnostic interpretation, then, the app will engage users and extend health care access to at‐risk patients.^27^

## CONFLICT OF INTEREST

The authors declare that they have no competing interests.

## AUTHOR CONTRIBUTIONS

(i) design and conduct of the study (Robinson, Turrisi), (ii) collection of data (Robinson), (iii) management, analysis and interpretation of data (Robinson, Turrisi, Mallett, Reavy), (iv) preparation of manuscript (Robinson), and (v) review, or approval of the manuscript (Mallett, Turrisi, Reavy).

## ETHICAL STATEMENT

All procedures performed in these studies involving human participants were in accordance with the ethical standards of the institution established by the Institutional Review Board of Northwestern University. This article does not contain any studies with animals performed by any of the authors.

Trial registration: clinicaltrials.gov Identifier NCT02854657.

## Data Availability

The data are available at Zenodo https://doi.org/10.5281/zenodo.3733197. Since the stage of melanoma resulting from SSE contains information that could compromise the privacy of research participants, these data are not publicly available.
